# Carbonized Polydopamine-Based Nanocomposites: The Effect of Transition Metals on the Oxygen Electrocatalytic Activity

**DOI:** 10.3390/nano13091549

**Published:** 2023-05-05

**Authors:** Jesús Cebollada, David Sebastián, María Jesús Lázaro, Maria Victoria Martínez-Huerta

**Affiliations:** 1 Instituto de Catálisis y Petroleoquímica, CSIC, Marie Curie 2, Cantoblanco, 28049 Madrid, Spain; jesus.cebollada@csic.es; 2 Instituto de Carboquímica, CSIC, Miguel Luesma Castán 4, 50018 Zaragoza, Spainmlazaro@icb.csic.es (M.J.L.)

**Keywords:** nitrogen-doped carbon, oxygen reduction reaction, oxygen evolution reaction, bifunctional catalyst, polydopamine, noble metal-free

## Abstract

The electrochemical oxygen reduction reaction (ORR) and oxygen evolution reaction (OER) are the most critical processes in renewable energy-related technologies, such as fuel cells, water electrolyzers, and unitized regenerative fuel cells. N-doped carbon composites have been demonstrated to be promising ORR/OER catalyst candidates because of their excellent electrical properties, tunable pore structure, and environmental compatibility. In this study, we prepared porous N-doped carbon nanocomposites (NC) by combining mussel-inspired polydopamine (PDA) chemistry and transition metals using a solvothermal carbonization strategy. The complexation between dopamine catechol groups and transition metal ions (Fe, Ni, Co, Zn, Mn, Cu, and Ti) results in hybrid structures with embedded metal nanoparticles converted to metal–NC composites after the carbonization process. The influence of the transition metals on the structural, morphological, and electrochemical properties was analyzed in detail. Among them, Cu, Co, Mn, and Fe N-doped carbon nanocomposites exhibit efficient catalytic activity and excellent stability toward ORR. This method improves the homogeneous distribution of the catalytically active sites. The metal nanoparticles in reduced (MnO, Fe_3_C) or metallic (Cu, Co) oxidation states are protected by the N-doped carbon layers, thus further enhancing the ORR performance of the composites. Still, only Co nanocomposite is also effective toward OER with a potential bifunctional gap (ΔE) of 0.867 V. The formation of Co-N active sites during the carbonization process, and the strong coupling between Co nanoparticles and the N-doped carbon layer could promote the formation of defects and the interfacial electron transfer between the catalyst surface, and the reaction intermediates, increasing the bifunctional ORR/OER performance.

## 1. Introduction

The increase in global energy consumption, the end of fossil fuels, climate change, and energy security have driven a massive effort to harvest energy from renewable sources. However, due to the intermittent energy generation from renewables, the development of devices capable of storing high energy density is in urgent demand [1,2]. Among them, fuel cells (FC), water electrolyzers (WE), or unitized regenerative fuel cells (URFC) have proven to be excellent options for a sustainable future. However, there are still challenges to overcome [3,4,5]. In these devices, the oxygen reduction reaction (ORR) and the oxygen evolution reaction (OER) take place at the oxygen electrode, and only in the case of URFC technology would bifunctional electrocatalysts be required [6,7,8]. However, oxygen reduction and evolution reactions generally require high overpotentials due to the slow kinetics and multielectron transfer pathways. Therefore, the electrode involves a catalyst based mainly on noble metals that represents a large portion of the cost of the device. Pt is the state-of-the-art catalyst for the ORR but is inefficient for the OER, and Ru and Ir oxides are commonly used for the OER but display low activity for ORR. However, the bifunctional activity of these noble metal mixtures is still not high enough, and their high cost and scarcity, joined with their low stability, limit their real applications [9,10].

Low-cost and highly abundant transition metals are attractive alternatives to replace noble metal-based catalysts, especially when an alkaline electrolyte is applied [11]. The alkaline medium favors the kinetics of the oxygen reactions (ORR/OER) compared to acidic conditions. In addition, it provides a less corrosive environment and higher durability for the catalysts. In particular, oxides and carbon-based noble metal-free catalysts are much more stable in alkaline medium [9,11,12,13,14,15,16,17]. Combining transition metals and heteroatom-doped carbon to form nanocomposites has gained considerable attention as multifunctional materials [18,19,20,21]. Nitrogen is the most extensively used dopant among non-metal heteroatoms such as B, N, S, and P due to its appropriate electronegativity. Moreover, N-doped carbon materials bonded to transition metals have been shown to enhance the catalytic activity and stability, likely due to the increased active sites, developed π-bonding networks in the carbon materials and improved electron donor ability [22,23,24,25,26].

The most widespread routes for synthesizing N-doped carbon materials are the direct reaction between carbon materials and nitrogen precursors or the carbonization of nitrogen-based precursors such as polymers [27,28,29,30,31]. Polydopamine (PDA) is a synthetic polymer with good affinity to almost any solid substrate due to the chemical bonding and physical interactions arising from its various functional groups (amines, imines or catechol) [32,33]. It is also one of the main pigments of eumelanin (natural melanin). Consequently, it has excellent biocompatibility, and exhibits many surprising properties in optics, electricity, and magnetism [34]. Polydopamine has similarities with mussel’s proteins, and much interest has come up in the study and application of PDA for coatings [35], environmental or catalytic applications [36], and energy storage and conversion [37]. The most popular method for preparing PDA is the oxidative polymerization of dopamine, as it can be carried out at room temperature in basic aqueous solutions, forming a PDA coating on various substrates. It is a complex process resulting in structural diversity and disorder, highly dependent on the monomer concentration, the type of buffer, the pH value, and the oxidant used [32,34,38]. PDA can also rapidly form in mildly acidic and neutral solutions using redox-active transition-metal ions [39]. In addition, this transition metal-mediated PDA polymerization also provides an attractive one-step approach for the synthesis of composite nanostructures [20,37,40]. For example, adding metal ions such as Ce^4+^ and Fe^3+^ to a dopamine solution accelerates the reaction at slightly acidic pH and shifts the effective range of dopamine oxidation and cyclization several pH units downward. However, Cu^2+^ is not a good enough oxidizing agent to initiate the process at a reasonable rate in acidic media and requires the presence of oxygen and chloride ions. In contrast, Cu^2+^ becomes a better oxidant in neutral and alkaline media than Ce^4+^ or Fe^3+^ [41]. The redox and complexation properties of the metals can also affect the final metal content. A large amount of metal occurs with moderately oxidizing metals making strong complexes with catecholate groups [39]. Furthermore, the presence of metal ions during the polymerization alters the structure and the properties of the PDA and other natural melanins. In the case of Cu^2+^, this effect is visible in the absorption spectra, in which the Cu-PDA absorbance profile is higher than PDA synthesized with only dissolved oxygen [42]. This opens up a wide range of possibilities for material modification, creating various novel hybrid materials with distinctive structures and characteristics.

In this work, we synthesized porous N-doped carbon nanocomposites by combining polydopamine (PDA) chemistry and transition metals (Fe, Ni, Co, Zn, Mn, Cu, Ti) using a solvothermal carbonization strategy. The influence of the transition metals on the structural, morphological, and electrochemical properties toward oxygen reduction and evolution reactions was analyzed in detail.

## 2. Materials and Methods

### 2.1. Materials and Chemicals

Cobalt (II) acetate tetrahydrate > 98.0%, copper (II) chloride dihydrate 99+%, titanium (IV) n-butoxide 99+%, and nickel (II) nitrate hexahydrate > 98.0% were purchased from Alfa Aesar (Kandel, Germany). Dopamine hydrochloride 100%, zinc (II) acetate dihydrate > 99.0%, iron (III) chloride 97%, potassium permanganate 99%, Nafion^®^ 5%wt solution, aqueous ammonia solution (30%), and sodium hydroxide 99.99% were purchased from Merck (Darmstadt, Germany). All aqueous solutions were prepared with ultrapure water (Milli-Q water, 18.2 MΩ cm). Platinum, nominally 20%, on carbon black and iridium (IV) oxide powder, 99%, purchased from Alfa Aesar, were used as benchmark catalysts.

### 2.2. Synthesis of Polydopamine-Based Composites

For each composite, 1.5 mmol of hydrochloric dopamine in ultrapure water (7.5 mL) was dropped to a solution of 3.0 mmol of the precursor metallic salt in anhydrous ethanol (95 mL) to obtain a molar ratio of 2:1 (dopamine: metal). Then, 3 mL NH_4_OH (30% solution) was added to the mixture to start the polymerization of the dopamine. After 45 min of stirring, the prepared mixture was transferred into a Teflon-lined stainless-steel autoclave and maintained at 150 °C for 4 h in a solvothermal process. The solid obtained was centrifuged and washed several times with ethanol and ultrapure water. Finally, the composite was dried at 70 °C and pyrolyzed at 800 °C for 2 h with a rate of 5 °C·min^−1^ under a nitrogen atmosphere. The composites synthesized were labeled as Ti-NC, Mn-NC, Fe-NC, Co-NC, Ni-NC, Cu-NC and Zn-NC. Additionally, a similar method was used to prepare carbonized polydopamine without metal. In this case, the mixture of dopamine solution and aqueous ammonia solution is stirred for 24 h instead of 45 min. The sample was labelled as PDA-NC.

### 2.3. Physicochemical Characterization

X-ray diffraction (XRD) diagrams of the composites were analyzed in X’Pert Pro PANalytical diffractometer (London, UK) θ-θ using CuKα1 (0.154056 nm). The diffractometer is equipped with a reaction chamber (Anton Paar XRK900) to obtain diffractograms while heating the powder samples in an N_2_ environment. The temperature ranged from 100 to 800 °C with 100 °C steps. Diffractograms were analyzed with the X’Pert HighScore Plus program. The metal content in the composites was quantified by inductively coupled plasma optical emission spectrometry (ICP-OES). The samples were dissolved in an acid solution, digested in a Multiwave 3000 Anton Paar and measured in PlasmaQuant^®^ PQ 9000 Analytik Jena (Jena, Germany). Elemental analyses (CHNS) were carried out in a LECO CHNS-932 microanalyzer. Adsorption isotherms were performed in Asap 2020, Micromeritics. The Brunauer–Emmet–Teller (BET) method was applied to calculate specific surface areas, and the Barret–Joyner–Halenda (BJH) method was applied to the curve of the nitrogen isotherms to obtain the pore size distributions. Isotherms were analyzed with MicroActive software. X-ray photoelectron spectroscopy (XPS) measurements were performed using a SPECS GmbH system with a UHV system with PHOIBOS 150 9MCD energy analyzer working with an Al Kα (hν = 1487 eV). The spectra were calibrated with the binding energy of the C-C bond of C1s at 284.6 eV. Transmission electron microscopy images were obtained in JEOL 2100F operating at 200 kV. Composites were powdered and dispersed in ethanol to be deposited on lacey copper (LC200-CU, EMS) or gold grid (LC200-AU-25, EMS).

### 2.4. Electrochemical Measurements

The electrochemical performance of the electrocatalysts was measured in a three-electrode cell at room temperature and controlled by an Autolab PGSTAT302. The ink catalyst solution was prepared by sonicating some catalyst powder in ethanol to obtain a concentration of 10 mg·mL^−^^1^ and 5 wt.% of Nafion solution. A quantity of 20 μL of catalytic ink was deposited in a glassy carbon disc (5 mm diameter, 0.196 cm^2^) of a rotating ring disc electrode (RRDE) to prepare the working electrode (WE). The collection efficiency of the RRDE platinum ring is 24.9%. As a counter electrode (CE), a gassy carbon rod was used, and a reversible hydrogen electrode (RHE) was employed as a reference electrode (RE). The electrolyte was 0.1 M NaOH aqueous solution. For all measurements, Ar was used to maintain an inert environment and for all OER experiments. O_2_ was employed to saturate the solution for ORR studies.

First, the electrolyte was deaerated with Ar for about 30 min. Then, an activation process was carried out for all composites based on 50 cyclic voltammetries (CV) between 0.05 and 1.2 V vs. RHE at a scan rate of 0.1 V·s^−^^1^. Then, blank voltammetry was recorded between 0.05 and 1.2 V vs. RHE at a scan rate of 0.02 V·s^−^^1^. The electrochemically active surface area (ECSA) was calculated as ECSA = C_dl_/C_s_ [43], considering the double layer capacitance (C_dl_) calculated by integrating the area in blank cyclic voltammetries, and assuming a specific capacitance (C_s_) of 0.4 F·m^−^^2^ [44]. For ORR measurements, two cycles of CV were performed in oxygen saturated solution between 1.2 V and 0.2 V vs. RHE at a scan rate of 0.005 V·s^−^^1^ and different electrode rotation speeds (rpm). The platinum ring was kept at 1.2 V vs. RHE. For OER measurements, 10 CV cycles were performed in saturated argon solution between 1.2 V and 1.8 V vs. RHE at a scan rate of 0.005 V·s^−^^1^ and 1600 rpm. The platinum ring was kept at 0.4 V vs. RHE. For the stability test on the best composite electrocatalyst, 10,000 cycles were performed between 0.6 and 1.2 V with a scan rate of 0.2 V·min^−^^1^ in Ar.

The current density, *j*, was calculated by dividing the current obtained, *i*, by the geometric area of the electrode (5 mm diameter, 0.196 cm^2^). The percentage of hydrogen peroxide production during the oxygen reduction reaction was calculated according to the following equation:

(1) $$\%H_{2}O_{2}=100\cdot \frac{2\cdot i_{ring}}{Ni_{disk}+i_{ring}}$$

where *i_disk_* and *i_ring_* are the absolute current from the RRDE disk and ring, respectively, and N is the collection efficiency for Pt ring of the RRDE. Tafel slope was used to evaluate the rate-determining step of the ORR. The Tafel relation can be expressed with Equation (2), where the applied potential (E) was corrected by the ohmic drop (iR, i is the current and R is the resistance), j_k_ represents the kinetic current density, b means the Tafel slope, and a is a constant:

E − iR = a + blog(j_k_) (2)

The determination of the number of exchanged electrons, *n*, was carried out by the Koutecky–Levich method:

(3) $$\frac{1}{j}=\frac{1}{j_{k}}+\frac{1}{0.62nFD_{O2}^{2/3}\nu^{-1/6}C_{O2}\omega^{-1/6}}$$

where *j_k_*, *ω* are the kinetic current density (mA/cm^2^) and the rotation rate of the electrode (rad/s), and *F*, *D_O_*_2_, *C_O_*_2_, υ are the Faraday constant (96485 F), the diffusion coefficient of oxygen (1.5 × 10^−^^5^ cm^2^/s), the concentration of oxygen in the electrolyte (mol/cm^3^), and the kinematic viscosity of the electrolyte (0.01 cm^2^/s), respectively.

## 3. Results and Discussion

### 3.1. Bifunctional Activity for ORR and OER

**Figure 1:**
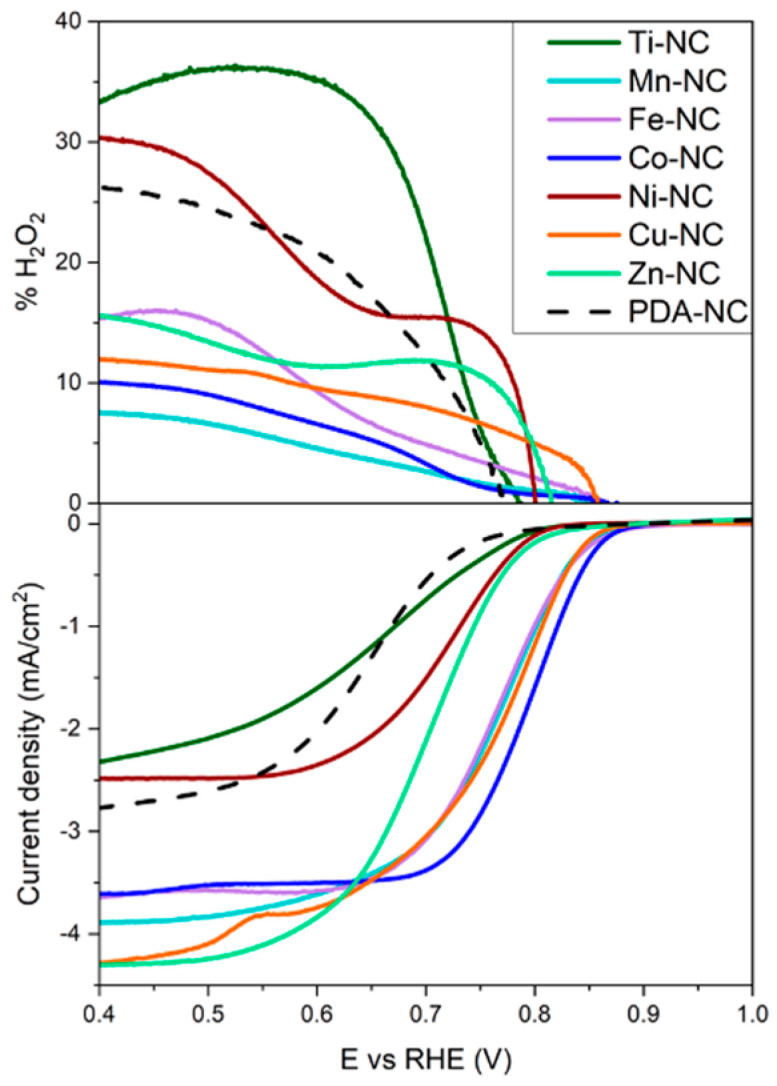
ORR polarization curves (below), hydrogen peroxide yield plots (above) for M-NC (M = Ti, Mn, Fe, Co, Ni, Cu, Zn and PDA) in oxygen saturated 0.1 M NaOH with a scanning rate of 0.005 V s^−1^, 1600 rpm.

CV measurements in an alkaline medium at a rotation rate of 1600 rpm were performed to investigate the bifunctional activity towards ORR and OER of the composites. Figure 1 displays the ORR polarization curves for the composites using an RRDE. The upper panel shows the Pt ring signal in terms of %H_2_O_2_ (the ring was kept at a constant potential of 1.2 V vs. RHE to detect the formation of H_2_O_2_), while the lower panel shows the disk signal. For the oxygen reduction reaction activity, the onset potential (E_onset_), half-wave potential (E_1/2_), electron transfer number (n), Tafel slope, and %H_2_O_2_ are essential indicators for evaluating the ORR performance (Table 1). As shown in Figure 1, the ORR activity is strongly influenced by the transition metal. The ORR onset and half-wave potential values of the polydopamine-based composites follow the order Co > Fe ≥ Mn ≥ Cu >> Zn > Ni > Ti > PDA. The Co-NC catalyst is the most active catalyst for the ORR, followed by Mn-NC, Fe-NC, and Cu-NC composites, in which comparable half-wave potential and electron transfer number are obtained. On the other hand, Ni-NC, Ti-NC, and PDA-NC catalysts show the largest onset potential and limiting current densities. The former can be related to the lower number of transferred electrons increasing the hydrogen peroxide production to values above 26% at 0.4 V vs. RHE (Table 1).

**Figure 2:**
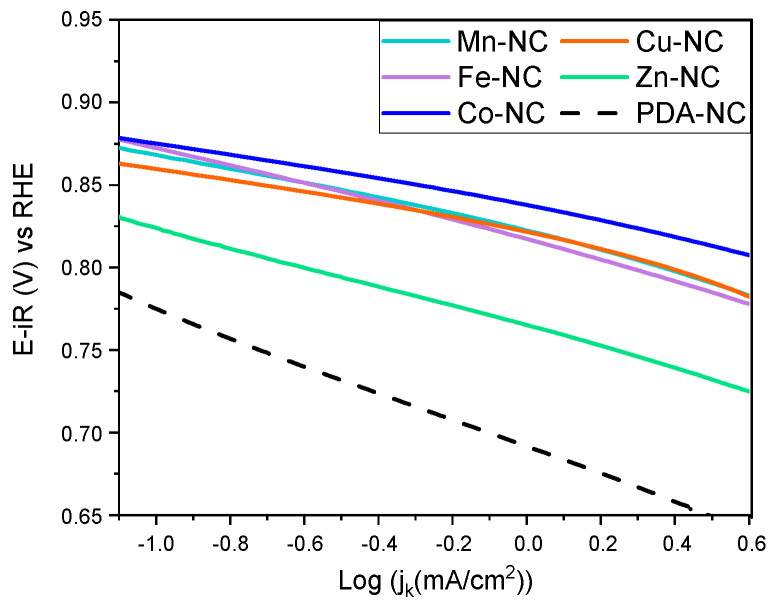
The Tafel plots in ORR for M-NC (M = Ti, Mn, Fe, Co, Ni, Cu, Zn and PDA).

The Tafel slope has been calculated at potential values within the kinetically controlled region of the ORR and compared with the theoretical values (Figure 2). In an alkaline medium, the following steps (Equations (4)–(7)) have been reported in the bibliography for the ORR mechanism [45]. Due to the multiple steps and the reaction intermediates formed (M denotes an empty site on the catalyst surface), the ORR mechanism is still under discussion.

M + O_2_ ⇄ MO_2_ (4)

MO_2_ + e^−^ ⇄ MO_2_^−^ (5)

MO_2_^−^ + H_2_O ⇄ MO_2_H + OH^−^ (6)

MO_2_H + e^−^ ⇄ MO + OH^−^ or MO_2_H + e^−^ ⇄ MOOH^−^ (7)

Co-NC and Cu-NC present the Tafel slope near 40 mV·dec^−^^1^, associated with the last step of the ORR (Equation (7)) being the rate-determining step (rds). Mn-NC, Fe-NC, Ni-NC and Zn-NC have similar values around 60 mV·dec-1. These values are ascribed to the proton exchange between water and the active center being the rds (Equation (6)). Ti-NC shows the highest slope, near 120 mV·dec^−^^1^, and the mechanism is limited by the formation of the MOO^−^ intermediate. The Tafel slope of PDA-NC is 82 mV·dec^−^^1^, and it is in between two steps, the formation of MO_2_^−^ (Equation (5)) and the proton exchange (Equation (6)) [45].

**Figure 3:**
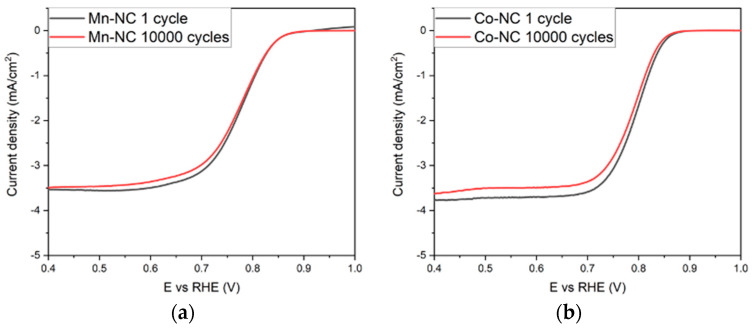

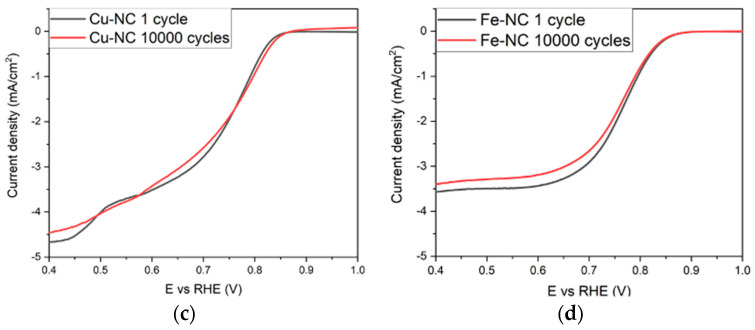
ORR polarization curves before (black) and after (red) 10,000 cycles of (**a**) Mn-NC, (**b**) Co-NC, (**c**) Cu-NC and (**d**) Fe-NC.

Other than high activity, durability experiments are another imperative parameter to evaluate the practicability of the catalysts. The stability tests were carried out for the most active catalysts, Mn-NC, Fe-NC, Co-NC, and Cu-NC, using 10,000 cycles voltammetries between 0.6 and 1.2 V vs. RHE with a scan rate of 0.2 V·min^−^^1^ in Ar. Two cyclic voltammetries were performed to determine the ORR activity before and after 10,000 cycles under the conditions described. The composites can maintain their ORR activity after the stability test, as shown in Figure 3. The catalytic activities are similar, and there are no indications of significant degradation, with a variation of the onset potential less than 0.005 V and the limiting current density below 0.1 mA·cm^−^^2^, demonstrating their excellent electrocatalytic durability.

**Figure 4:**
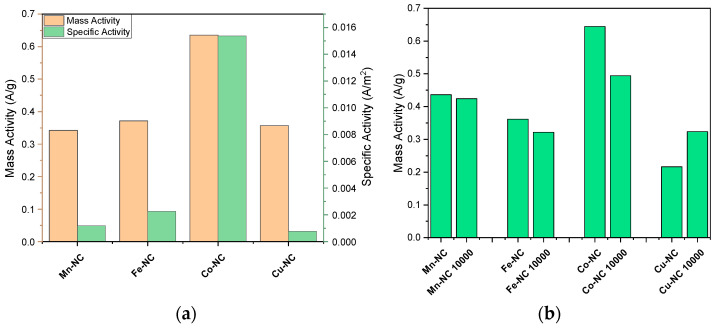
ORR specific activity and mass activity of Mn-NC, Fe-NC, Co-NC and Cu-NC (**a**), and ORR mass activity of the same catalysts before and after stability test (10,000 cycle voltammetries) (**b**) at 0.835 V vs. RHE.

To further explore the intrinsic electrochemical characteristics of the catalysts, the electrochemically active surface area (ECSA) was calculated by determining the electrochemical double-layer capacitance using the CV technique (Table 1) [46]. It is generally acknowledged that ECSA has a linear relationship with the electric double layer (EDL) due to the interfacial charging process [47]. The corresponding CV curves and the capacitance values are presented in Figure S4. As shown in Table 1, the Cu-NC catalyst offers the highest ECSA providing abundant active sites for the reactants. In contrast, the Co-NC composite shows the lowest value of ECSA but the higher specific activity at 0.835 V (Figure 4a).

The mass activity was also considered when determining the performance of the electrocatalysts. The current response normalized by the electrocatalyst mass is represented by mass activity (A g^−1^), which was calculated using the following equation: mass activity = j/m [46] where j (mA cm^−2^) refers to the current density at a given potential and m is the mass loading of catalyst (mg cm^−2^) on the electrode. Figure 4a shows the mass activity and specific activity of the most active catalysts, Mn-NC, Fe-NC, Co-NC and Cu-NC at 0.835 V vs. RHE. Accordingly, the Co-NC electrocatalyst exhibits the highest ORR specific and mass activity. Furthermore, Figure 4b shows mass activity before and after the stability test of the composites. A slight decrease in Mn and Fe composites are observed, while Co-CN shows a more significant loss of mass activity. Only Cu-NC displays a slight increase.

**Figure 5:**
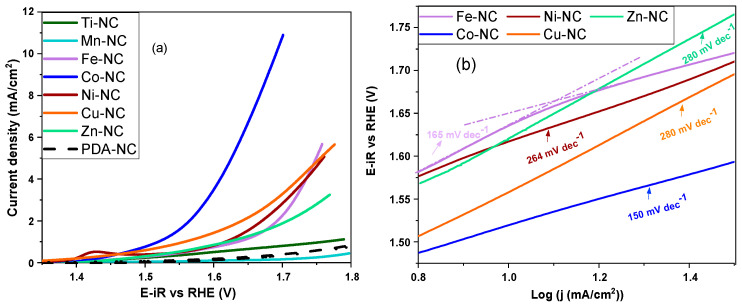
OER polarization curves for M-NC (M = Ti, Mn, Fe, Co, Ni, Cu, Zn and PDA) in 0.1 M NaOH with a scanning rate of 5 mV s^−1^ at 1600 rpm (**a**); Tafel plots derived from the OER polarization curve (**b**).

The effect of the transition metal on the OER electrocatalytic activity is reported in Figure 5a. It is observed that Co-NC is the most active composite, showing an overpotential value of 0.460 V at 10 mA·cm^−2^ current density. There is a remarkable change in the OER activity when comparing Co-NC with the rest of the composites that cannot reach 10 mA·cm^−2^. The low activity of Ti-NC and Mn-NC is noticeable, similar to PDA-NC. Therefore, this synthesis method does not contribute to forming Ti and Mn active sites in the oxygen evolution reaction. Moreover, aiming to evaluate the reaction kinetics of the more active composites, the Tafel plots are represented in Figure 5b. Performing the same study as for the ORR, the values obtained are higher than the theoretical values attributed to the reaction mechanisms of the OER in alkaline media [45]. This may be due to other factors affecting the Tafel slope, such as parallel reaction to the OER, adsorbates on the catalyst surface, or surface–electrolyte interaction [48,49]. Kapalka et al. found high Tafel slopes in boron-doped diamond electrodes resulting from the surface redox couples/functional groups, which act as a barrier for OER [48]. Furthermore, Doyle et al. pointed out that an increase in the Tafel slope is not necessarily mechanistically significant. A continuous increase in the Tafel slope with applied potential may also be due to a reduction in the effective surface area of the electrode with increasing gas evolution at the higher applied potentials [49].

**Figure 6:**
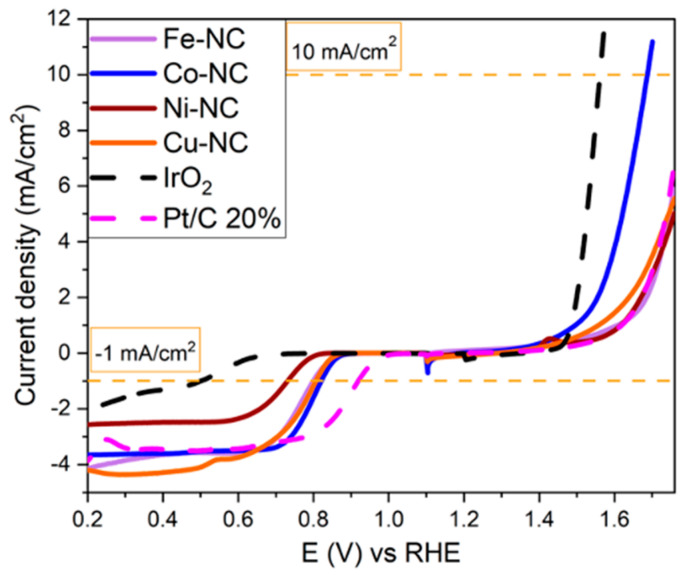
ORR and OER polarization curves of Fe-NC, Co-NC, Ni-NC and Cu-NC compared to Pt/C and IrO_2_.

The above electrochemical results for ORR and OER demonstrate that the Co-NC composite is the most active catalyst in both reactions. Figure 6 shows the polarization curves of the best PDA-based composites (Co-NC, Fe-NC, Ni-NC and Cu-NC) to better compare the bifunctional activity. IrO_2_ and Pt/C have been added as commercial benchmarking catalysts. The potential bifunctional gap (ΔE), or difference in potential between the OER current density at 10 mA·cm^−2^ and the ORR current density at −1 mA·cm^−2^, are 0.867 V and 1.061 V for Co-NC and IrO_2_, respectively. The smaller the potential gap, the better the bifunctional ORR/OER behavior. These results imply that the most active catalysts prepared using this method have a higher grade of bifunctionality than IrO_2_, even Co-NC outperforming the bifunctional behavior of Pt/C.

### 3.2. Structural Characterization of the Composites

**Figure 7:**
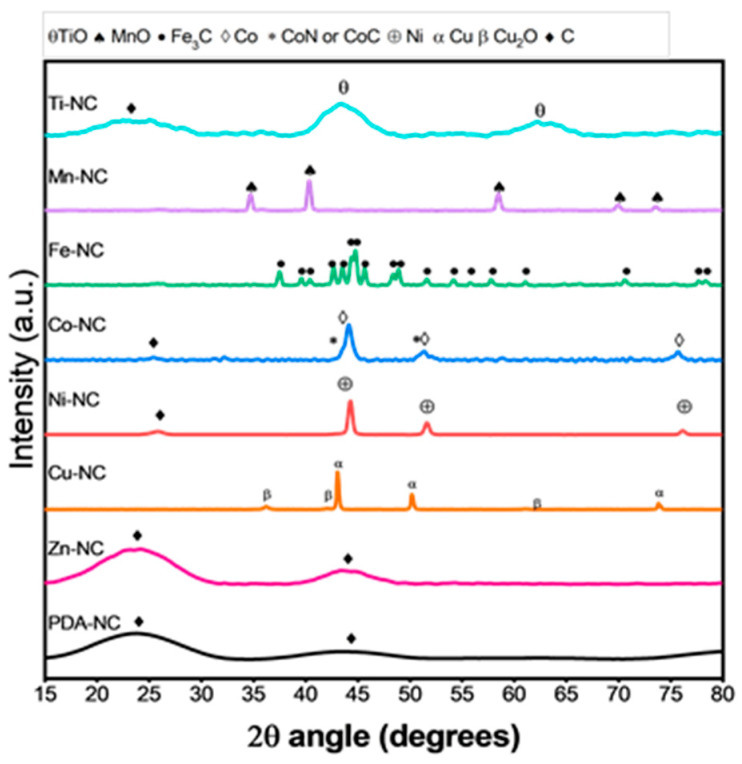
XRD patterns of M-NC (M = Ti, Mn, Fe, Co, Ni, Cu, Zn or PDA).

Figure 7 shows the XRD patterns of the PDA composites. PDA-NC and Zn-NC display two broad diffraction peaks at 2θ around 25° and 44° attributable to the (002) and (100) diffractions of graphitic carbon (JCPDS, #13-0148). Both composites show similar XRD patterns since Zn evaporates at high temperatures during pyrolysis, as has been further confirmed by ICP-OES (see Table S1). The graphitic carbon can also be observed in Ti-NC composite, with two additional broad peaks associated with titanium (II) oxide (JCPDS #72-0020). Interestingly, the presence of polydopamine prevents the formation of TiO_2_ during the synthesis process keeping titanium in a higher reduction state (TiO). For the Mn-NC composite, the diffraction peaks can be ascribed to manganese (II) oxide (Manganosite, JCPDS #06-0592). Co-NC composite shows diffraction peaks corresponding to metallic cobalt with face-centered cubic structure (JCPDS #15-0806), and the broadening in the cobalt peaks can be attributed to cobalt nitride (JCPDS #41-0943). Ni-NC displays the characteristic diffraction peaks of metallic Ni (JCPDS #04-0850), and Cu-NC composite shows diffraction peaks assigned to metallic Cu (JCPDS #04-0836) and copper (I) oxide (cuprite, JCPDS #01-1142). Fe-NC displays diffraction peaks corresponding to iron carbide (cohenite, JCPDS #72-1110).

**Scheme 1:**
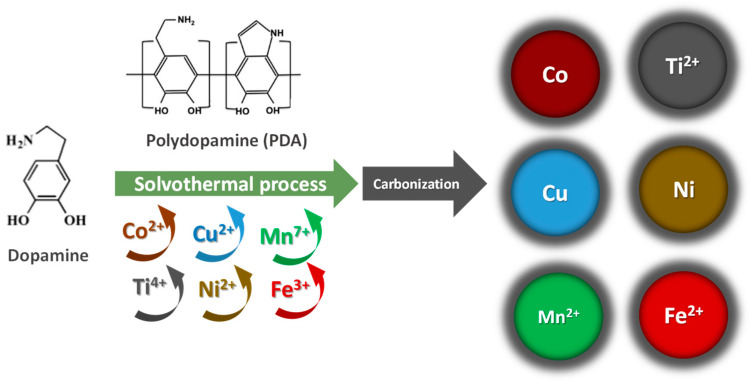
Preparation of M-NC (M = Ti, Mn, Fe, Co, Ni, Cu) nanocomposites.

These results indicate the reduction capability of the catechol moiety during the dopamine polymerization process on these transition metals to obtain hybrid nanostructures [32,50]. Even though the synthesis conditions are identical for the formation of the hybrid nanostructures, the presence of different metallic ions influences the intermediates in dopamine polymerization, and in turn the oxidation speed and the size distribution of the polydopamine colloids [32,51]. In agreement with the XRD results, carbonization of hybrid nanostructures with embedded transitional metal species, such as, Co (II), Cu (II), Ti (IV), Ni (II), Fe(III), and Mn (VII)), are converted to metal–PDA composites with lower oxidation states, such as, Co, Cu, Ti (II), Ni, Fe(II) and Mn(II)), respectively (Scheme 1).

**Figure 8:**
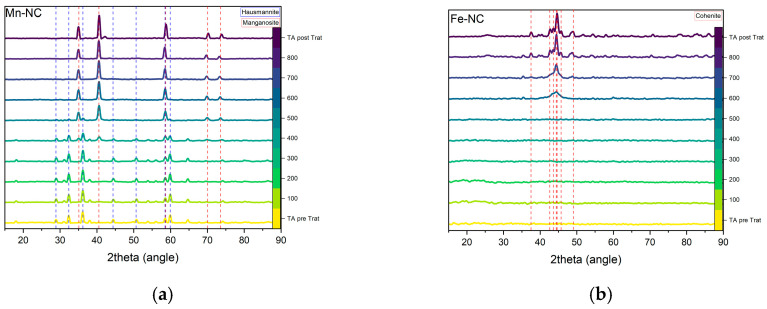

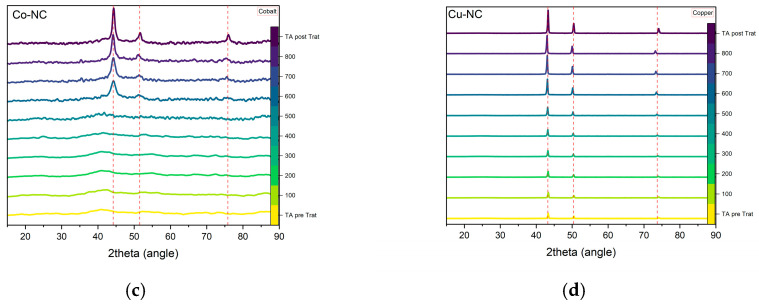
Temperature-dependent in situ XRD results for (**a**) Mn-NC; (**b**) Fe-NC; (**c**) Co-NC; (**d**) Cu-NC composites under N_2_ atmosphere. XRD patterns in TA pre Trat and TA post Trat are obtained at ambient temperature before (pre Trat) and after (post Trat) in-situ measurements.

To study the phase evolution of the most active ORR electrocatalysts (Mn-CN, Cu-CN, Co-CN and Fe-CN) in situ X-ray diffraction under N_2_ atmosphere after the solvothermal process was investigated (Figure 8). For the Mn-NC composite, the diffraction pattern of manganese (II, III) oxide (Hausmannite, JCPDS #24-0734) is observed after solvothermal treatment. As the temperature rises above 300 °C, hausmannite peaks decreased, and diffraction peaks assigned to manganese (II) oxide were formed. Above 600 °C, only manganese (II) oxide could be observed (Figure 8a). For the Fe-NC sample, the two broad diffraction peaks assigned to graphitic carbon is the only phase followed until the formation of Fe_3_C species above 600 °C (Figure 8b). In Figure 8c, the appearance of cobalt metal particles is observed between 500 and 600 °C. Unlike the other composites, metallic copper is the unique species detected after solvothermal treatment up to 800 °C (Figure 8d). This indicates the easy reduction of Cu (II) in the presence of catechol during polymerization.

**Figure 9:**
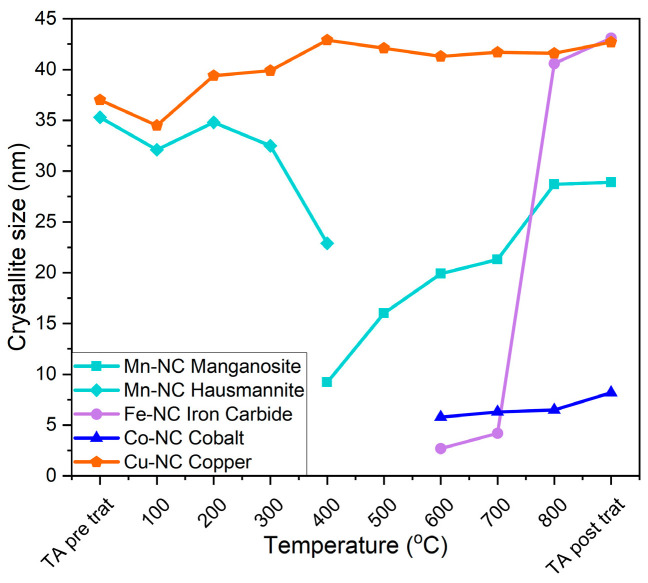
The crystallite size of the main composite species at different carbonization temperatures.

The Debye–Scherrer method was used to analyze the crystallite size of the main crystal phases with the carbonization temperature in Mn-CN, Fe-CN, Co-CN, and Cu-CN composites (Figure 9). The (211) reflection of hausmannite and (200) reflection of manganosite were chosen to analyze the Mn-CN composite. Hausmannite has a crystallite size of 35 nm, which decreases to 23 nm with temperatures up to 400 °C, overlapping with the formation of manganese (II) oxide with a crystallite size of 10 nm. The temperature increase causes an increment in crystallite size up to 27 nm. The (031) reflection of Fe_3_C was used to analyze the Fe-NC composite. An abrupt increase in crystallite size from 3 to 40 nm is observed as the temperature rises from 700 to 800 °C. The (111) reflection of Co was used to analyze the Co-CN composite. Crystallite size between 6–8 nm is maintained at all temperatures. The (111) reflection of Cu is used to analyse the Cu-CN sample. The crystallite size range between 35 and 43 nm is maintained throughout the carbonization process.

**Figure 10:**
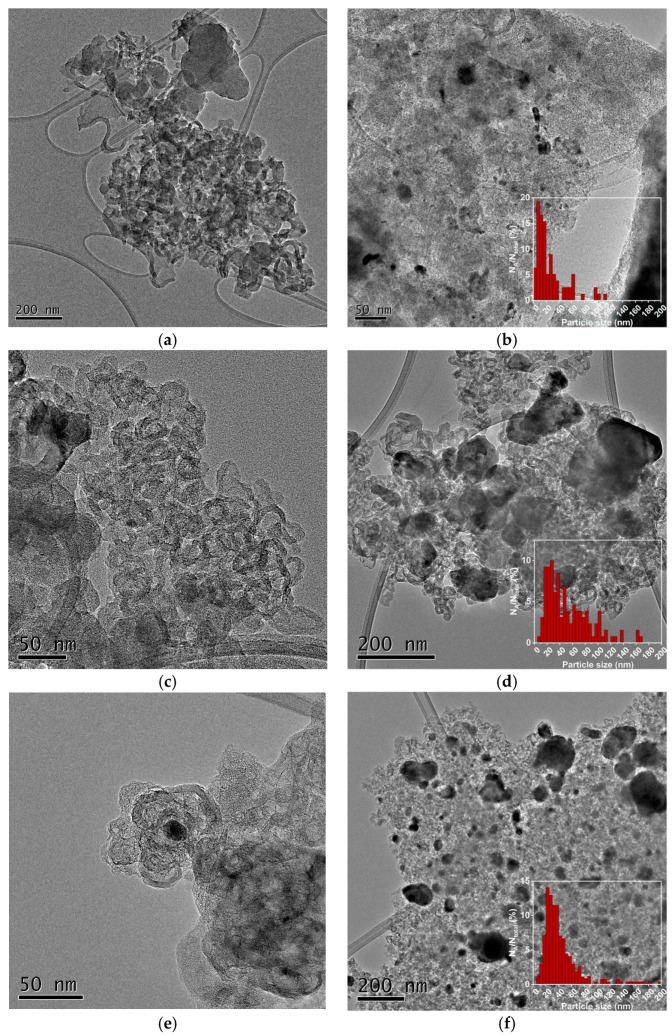

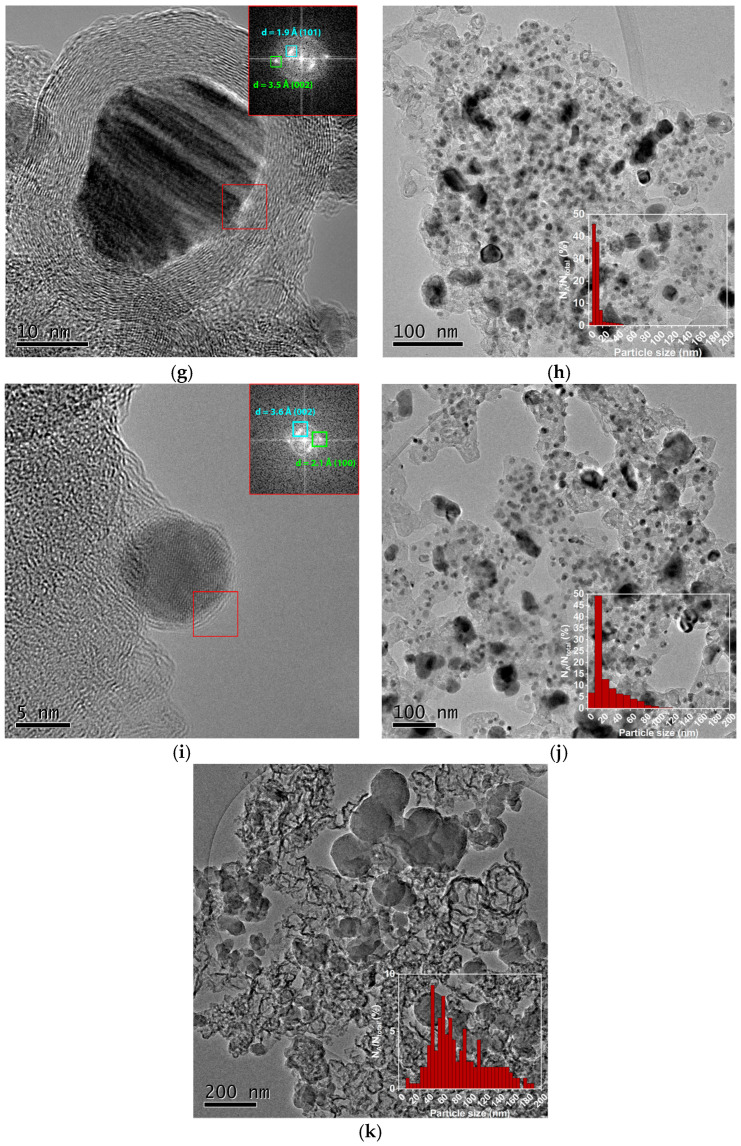
TEM images and particle size distributions of (**a**) Zn-NC; (**b**) Ti-NC; (**c**,**d**) Mn-NC; (**e**,**f**) Fe-NC; (**g**,**h**) Co-NC; (**i**,**j**) Ni-NC; (**k**) Cu-NC.

Figure 10 shows the TEM images of the M-NC composites. A significant effect of the transition metal on the structure and dispersion of the composites is observed. Except for Ti-NC (Figure 10b), in the rest of the carbonized polydopamine-based composites, the nitrogen-doped carbon gives rise to deformed spherical nanostructures. Zn-NC composite confirms the formation of distorted structures in the absence of metal (Figure 10a). Mn, Fe, and Cu generate composites with heterogeneous metallic particle sizes, in which the aggregation of nanoparticles is apparent during the carbonization process (Figure 10c–f,k). Co- and Ni-related catalysts show carbonized PDA coating of the metallic nanoparticles with different thicknesses, those of Ni-NC being thinner (Figure 10g,i). The distance between carbon layers surrounding the Co and Ni particles is 3.49 ± 0.1 and 3.64 ± 0.1 Å, respectively, and can be attributed to the plane (002) of the distorted graphite. Other carbon planes are observed above the particles, (101) for Co-NC (d-spacing of 1.89 Å) and (100) for Ni-NC (d-spacing of 2.11 Å). These planes show the partial graphitization of the carbon during pyrolysis. The carbon layers avoid metal aggregation keeping particle size below 20 nm (Figure 10h,j).

These differences are associated with the two processes that take place during the synthesis of composites, the coordination between the metal and the polymer during hydrothermal treatment and the carbonization process. The complexation between the PDA catechol groups and transitional metal species has been widely studied [32]. Depending on the transition metal coordination number, their complexation with PDA and covalent polymerization of dopamine can occur simultaneously, resulting in hybrid nanostructures with embedded transitional metal species. Carbonization converts the PDA layer to a highly graphitized N-doped carbon layer, and a heterogeneous composite is obtained.

### 3.3. Surface Characterization

**Figure 11:**
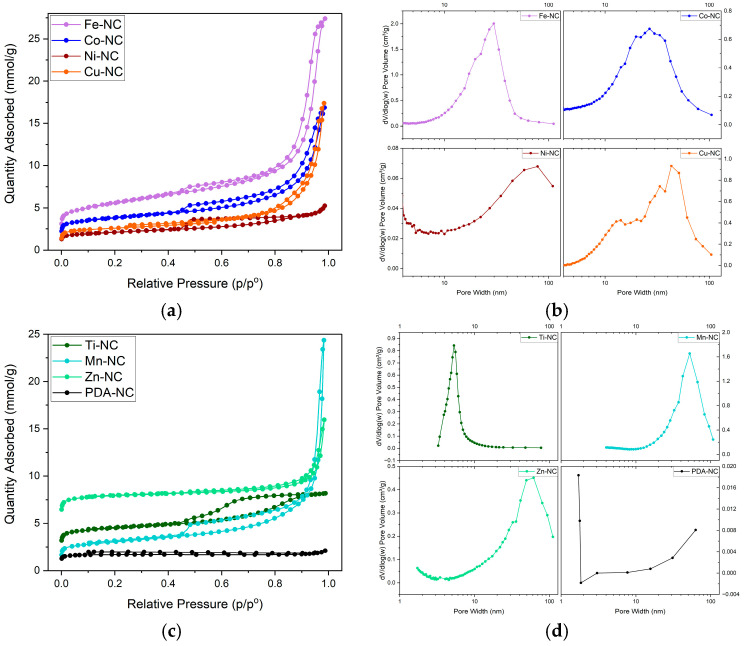
N_2_ adsorption-desorption isotherms and the pore size distribution: (**a**,**b**) Fe-NC, Co-NC, Ni-NC and Cu-NC; (**c**,**d**) Ti-NC, Mn-NC, Zn-NC and PDA-NC.

The textural properties of the composites were characterized by nitrogen adsorption–desorption isotherms (Figure 11). The addition of metal to the polymer increases the BET surface area in the following order Zn > Fe > Ti > Co > Mn > Cu > Ni > PDA (Table 2). The two metal-free composites, PDA-NC and Zn-NC, with the lowest surface area of 151 m^2^·g^−1^ and the largest surface area of 714 m^2^·g^−1^, respectively, showed type I isotherms. PDA-NC exhibited high microporous content, while an H4 hysteresis loop was observed in Zn-NC, manifesting the coexistence of microporous, mesoporous, and macroporous structures. The rest of the catalysts displayed type IV isotherms. The marked hysteresis loop in the 0.4–0.9 P/P^0^ range is characteristic of mesoporous materials, which could also be observed in the pore size distribution (Figure 11B,D). The porosity of these composites is related to the metal added to the polymer, obtaining different pore-size distributions. Ti-NC has homogeneous and narrow pore-size distributions centered at 5 nm. Ni-NC has macropores centered at 78 nm. Fe-NC, Co-NC, and Cu-NC have mesopores with average pore sizes of 30, 26 and 43 nm, respectively. Mn-NC is similar to the latter ones but with a more significant contribution of macropores. Zn-NC shows macroporous distribution around 62 nm.

**Figure 12:**
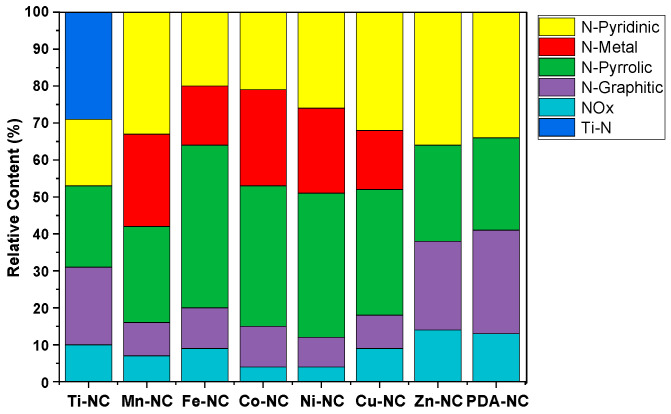
The relative content of nitrogen species of M-NC catalyst M-NC (M = Ti, Mn, Fe, Co, Ni, Cu and Zn).

X-ray photoelectron spectroscopy (XPS) was performed to study the surface chemical composition of the composites and the nitrogen species distribution. All of them show the characteristic peaks of C1s, O1s, and N1s, and the corresponding metals (Ti2p, Mn2p, Co2p, Cu2p, Ni2p, Fe2p and Zn2p). To better understand the chemical environment of doped nitrogen, the N1s spectrum has been deconvoluted into five subpeaks (Figure S1). The occurrence peaks at ca. 398, 400, 402, and 403 eV refer to N bonded to carbon and corresponding to N-pyridinic, N-pyrrolic, N-graphitic and NOx species, respectively [52,53,54,55]. A peak corresponding to nitrogen coordinated with metal (N-metal) is observed at ca. 399 eV in Mn-NC, Fe-NC, Co-NC, Ni-NC, and Cu-NC composites [56,57]. In addition, a peak at 396.3 eV is evident for Ti-NC, associated with Ti-N interactions [19]. The relative content of the nitrogen species was calculated and depicted in Figure 12. N-pyridinic, N-graphitic and nitrogen-coordinated metals have been identified as important chemical sites for ORR and OER [58,59,60]. However, it should be noted that the order of these N species is inconsistent with the order of ORR and OER performance of the composites. For example, Zn-NC and PDA-NC have similar content of nitrogen components but exhibit different electrocatalytic activities. This can also be attributed to the evaporation of Zn, which generates defects and a larger porous structure in Zn-NC, increasing the activity towards OER and ORR [61]. The incorporation of the metal results in an apparent decrease of the N-graphitic species and a similar distribution of the different species among the various metal composites.

The chemical state of metal has been evaluated from high-resolution XPS, Ti2p, Mn2p, Fe2p, Co2p, Ni2p, Cu2p, and Zn2p (Figure S2). O1s XPS spectra of all composites are shown in Figure S3. This information must be interpreted while considering its limitations since the catalyst’s oxidation state probably changes during the electrochemical process. Table 2 shows the surface nitrogen and metal content. Compared with the bulk composition (Table S1), the metal loading on the surface is rather low (1.4–7.3 wt.%), except for Ti-NC (21 wt.%). That means that nanoparticles of metals such as Ni, Co, Cu, Mn, and Fe are mainly distributed inside the porous carbonized structure or coated by carbon layers, as revealed by the TEM images. Surface nitrogen was detected in all composites, and its content varies from 1.5 to 8.6 wt.%. It manifests how the different transition metals can fix different amounts of nitrogen during carbonization, resulting in materials with an unlike surface chemical composition.

We cannot simply correlate the ORR/OER performance with the surface and total N content or any single factor. The electroactivity of the composites can be affected by the geometrical location of the active site, as well as the nearest carbon neighbor’s and the oxidation state, active N content, pore structure, defects, and other qualities. The carbonization of the polyamide under the synthesis conditions used in this work has allowed Co-NC, Fe-NC, Cu-NC, and Mn-NC composites to exhibit unusual activities and stabilities towards ORR. The metal nanoparticles in reduced (MnO, Fe_3_C) or metallic (Cu, Co) oxidation states are protected by the N-doped carbon layers, thus further enhancing the stability of the composites. Among them, Co nanocomposite is also effective toward OER. It can be attributed to an increased mass and specific activity. The unusual ORR/OER activity of Co-N-Carbon based structures is already known [7,20,62]. Density functional theory (DFT) calculations demonstrated that nitrogen doping and the electron transfer from the embedded metal nanoparticle to the outer carbon layer could decrease the local work function on the carbon surface, increasing catalytic activity [62]. This simple method can ensure the homogeneous distribution of catalytically active and improves the intrinsic catalytic activity and mass activity of the composites by engineering N-dopant and interfaces.

## 4. Conclusions

The carbonization of the coated polydopamine on Ti, Mn, Fe, Co, Ni, Cu, and Zn metal ions leads to N-doped composites with a variety of structures, morphologies, surface areas, chemical compositions, and electrochemical ORR/OER activity, evidencing the critical role played by the transition metal during the solvothermal carbonization strategy in similar operating conditions. Co-NC, Fe-NC, Cu-NC, and Mn-NC composites exhibit unusual activities and stabilities towards ORR. This method improves the homogeneous distribution of the catalytically active sites. It promotes the formation of N-doped carbon layers coated on the metal nanoparticles, thus further enhancing the stability of the composites. In addition, Co-NC nanocomposite is also effective toward OER. The formation of Co-N active sites and the strong coupling between Co nanoparticles and the N-doped carbon layer could significantly promote the formation of defects and the interfacial electron transfer between the catalyst surface and the reaction intermediates, drastically boosting the bifunctional ORR/OER performance.

## Figures and Tables

**Table 1:** ORR kinetic data for M-NC (M = Ti, Mn, Fe, Co, Ni, Cu, Zn and PDA) and electrochemical surface area of the catalysts (ECSA).

Catalyst	E_onset_ (V vs. RHE) at 0.1 mA·cm^−2^	E_1/2_ (V vs. RHE)	J_lim_ (mA·cm^−2^) at 0.4 V vs. RHE	n (e^−^)	J_k_ (mA·cm^−2^) at 0.79 V vs. RHE	Tafel Slope (mV·dec^−1^)	% H_2_O_2_ (at 0.4 V vs. RHE)	ECSA (m^2^·g^−1^)
Ti-NC	0.79	0.66	2.3	2.5	0.1	111	33	204
Mn-NC	0.86	0.76	3.9	3.0	1.5	53	8	288
Fe-NC	0.87	0.77	3.6	3.7	1.3	59	15	164
Co-NC	0.87	0.79	3.6	3.4	2.7	43	10	41
Ni-NC	0.80	0.72	2.5	2.6	0.17	59	30	52
Cu-NC	0.86	0.76	4.3	3.5	1.8	42	12	464
Zn-NC	0.82	0.67	4.3	3.2	0.25	61	16	222
PDA-NC	0.77	0.65	2.8	3.3	0.11	82	26	52

**Table 2:** Textural values from the nitrogen physisorption analysis and surface composition by XPS of the composites.

Catalyst	BET-Specific Surface Area (m^2^·g^−1^)	Micropore Area (m^2^·g^−1^)	External Area (m^2^·g^−1^)	Surface Metal (wt.%)	Surface Nitrogen (wt.%)
Ti-NC	387	291	96	21	2.0
Mn-NC	247	123	124	7.3	4.2
Fe-NC	447	207	240	1.5	2.5
Co-NC	308	165	143	1.4	1.5
Ni-NC	170	94	76	2.7	2.0
Cu-NC	216	129	87	2.9	8.6
Zn-NC	714	665	49	1.9	3.7
PDA-NC	151	150	1	-	6.4

## Data Availability

Not applicable.

## Supplementary Materials

The following supporting information can be downloaded at: https://www.mdpi.com/article/10.3390/nano13091549/s1, Figure S1: N 1s XPS spectra of (a) Ti-NC; (b) Mn-NC; (c) Fe-NC; (d) Co-NC; (e) Ni-NC; (f) Cu-NC; (g) Zn-NC; (h) PDA-NC; Figure S2: (a) Ti2p XPS spectra of Ti-NC; (b) Mn2p XPS spectra of Mn-NC; (c) Fe2p3/2 XPS spectra of Fe-NC; (d) Co2p3/2 XPS spectra of Co-NC; (e) Ni2p3/2 XPS spectra of Ni-NC; (f) Cu2p3/2 XPS spectra of Cu-NC; (g) Zn2p3/2 XPS spectra of Zn-NC; Figure S3: O 1s XPS spectra of (a) Ti-NC; (b) Mn-NC; (c) Fe-NC; (d) Co-NC; (e) Ni-NC; (f) Cu-NC; (g) Zn-NC; (h) PDA-NC; Figure S4. CV profile of the as-synthesized electrocatalysts measured in 0.1 M NaOH at a scan rate of 20 mV s^−1^; Table S1: Chemical analysis of M-NC (M = Ti, Mn, Fe, Co, Ni, Cu, Zn and PDA) (wt%).
