# Supplementary material for: Carbonized Polydopamine-Based Nanocomposites: The Effect of Transition Metals on the Oxygen Electrocatalytic Activity
Source: Nanomaterials (Basel). 2023 May 5;13(9):1549. doi: 10.3390/nano13091549 (PMC10180844; doi:10.3390/nano13091549)
Supplement: Supplementary file 1 [file nanomaterials-13-01549-s001.zip › nanomaterials-2348857-supplementary.pdf]

Supplementary Materials

Carbonized Polydopamine-Based Nanocomposites: The Effect of Transition Metals on the Oxygen Electrocatalytic Activity

Table S1. Chemical analysis of M-NC (M = Ti, Mn, Fe, Co, Ni, Cu, Zn and PDA) (wt%)

**Catalyst**	**C**	**N**	**M**
**Ti-NC**	54	2.7	18
**Mn-NC**	46	3.4	35
**Fe-NC**	63	1.7	46
**Co-NC**	57	2.0	42
**Ni-NC**	63	1.6	33
**Cu-NC**	52	7.2	39
**Zn-NC**	85	4.4	1
**PDA-NC**	79	6.2	-

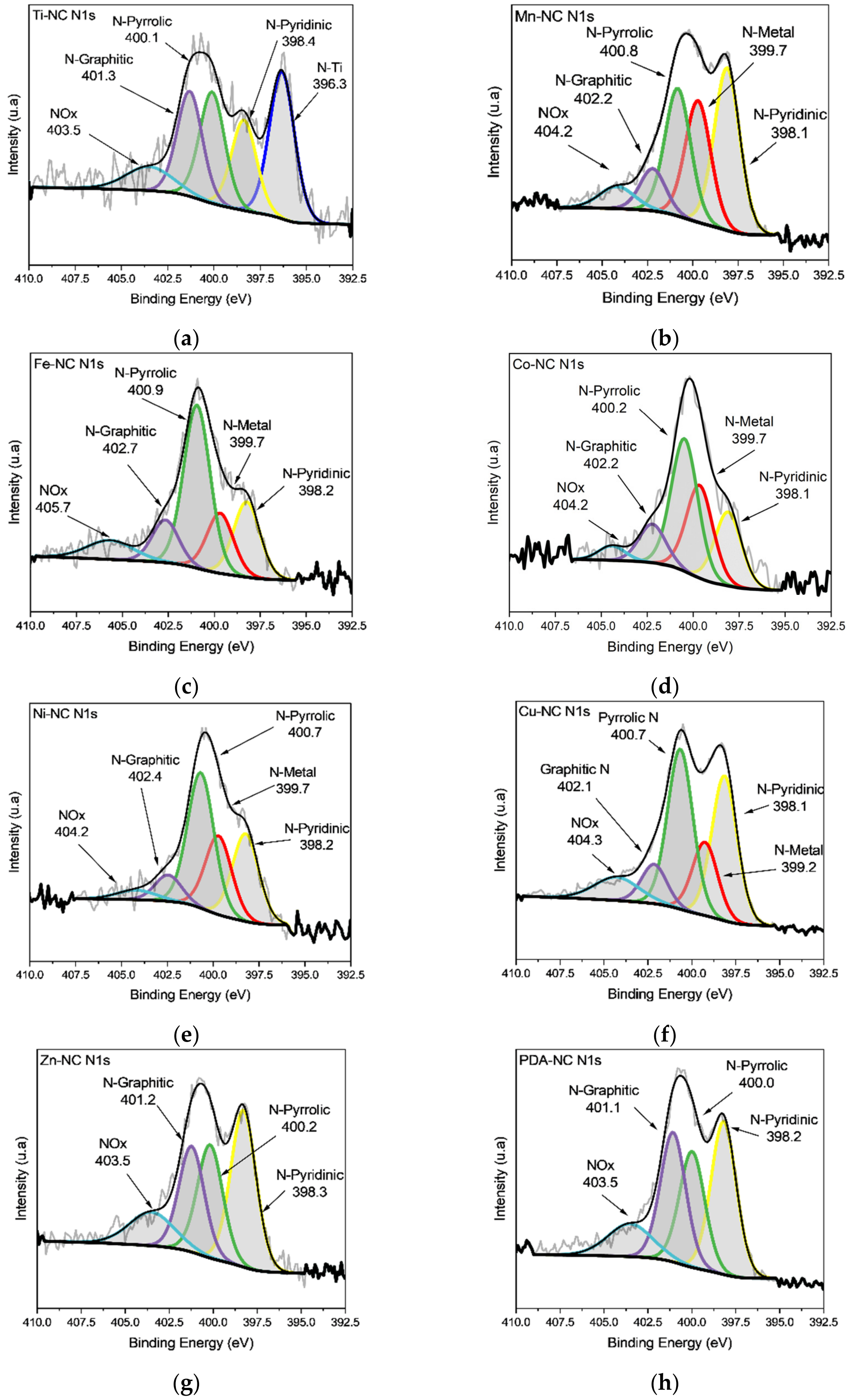

Figure S1. N 1s XPS spectra of (**a**) Ti-NC; (**b**) Mn-NC; (**c**) Fe-NC; (**d**) Co-NC; (**e**) Ni-NC; (**f**) Cu-NC; (**g**) Zn-NC; (**h**) PDA-NC

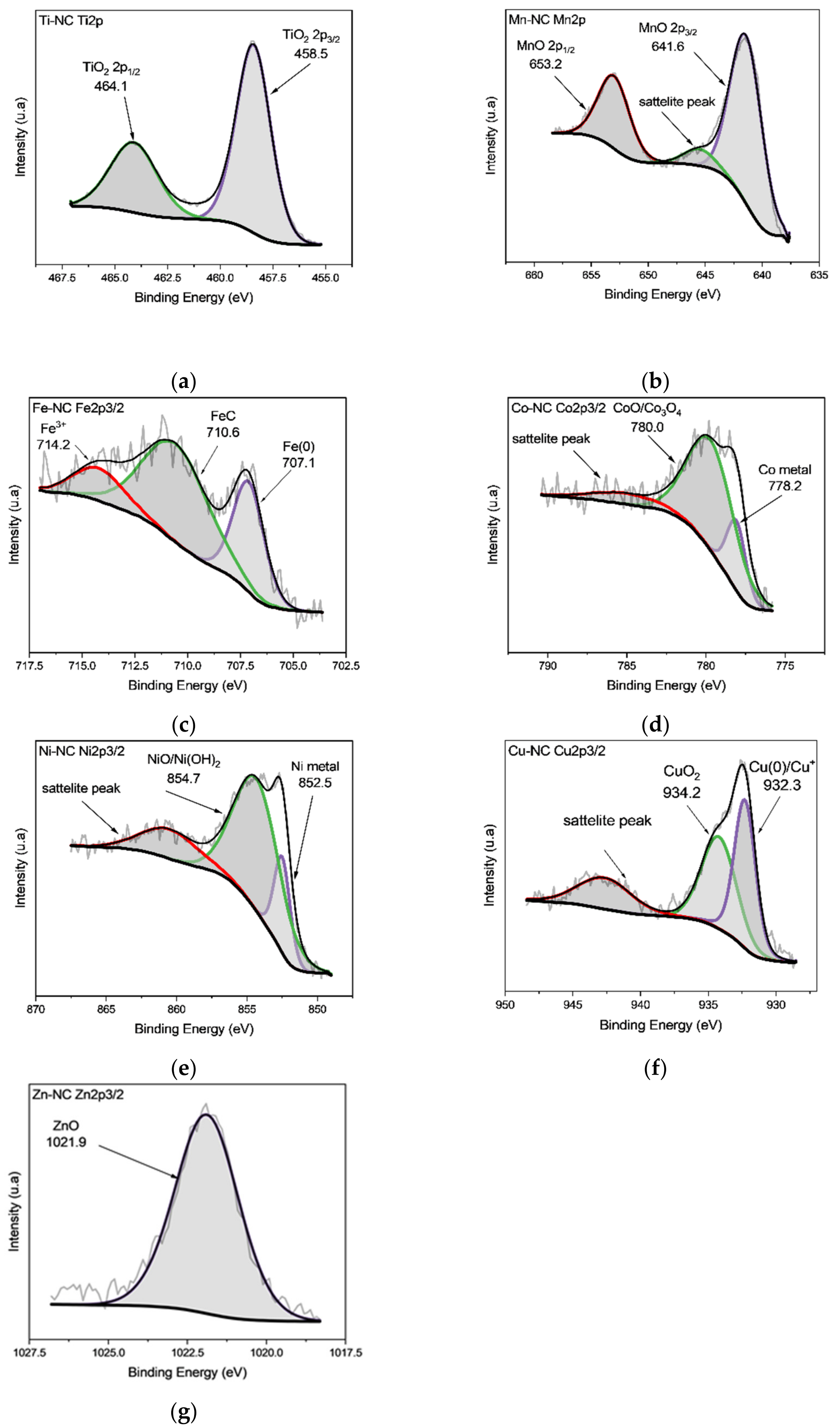

Figure S2. (**a**) Ti2p XPS spectra of Ti-NC; (**b**) Mn2p XPS spectra of Mn-NC; (**c**) Fe2p3/2 XPS spectra of Fe-NC; (**d**) Co2p3/2 XPS spectra of Co-NC; (**e**) Ni2p3/2 XPS spectra of Ni-NC; (**f**) Cu2p3/2 XPS spectra of Cu-NC; (**g**) Zn2p3/2 XPS spectra of Zn-NC

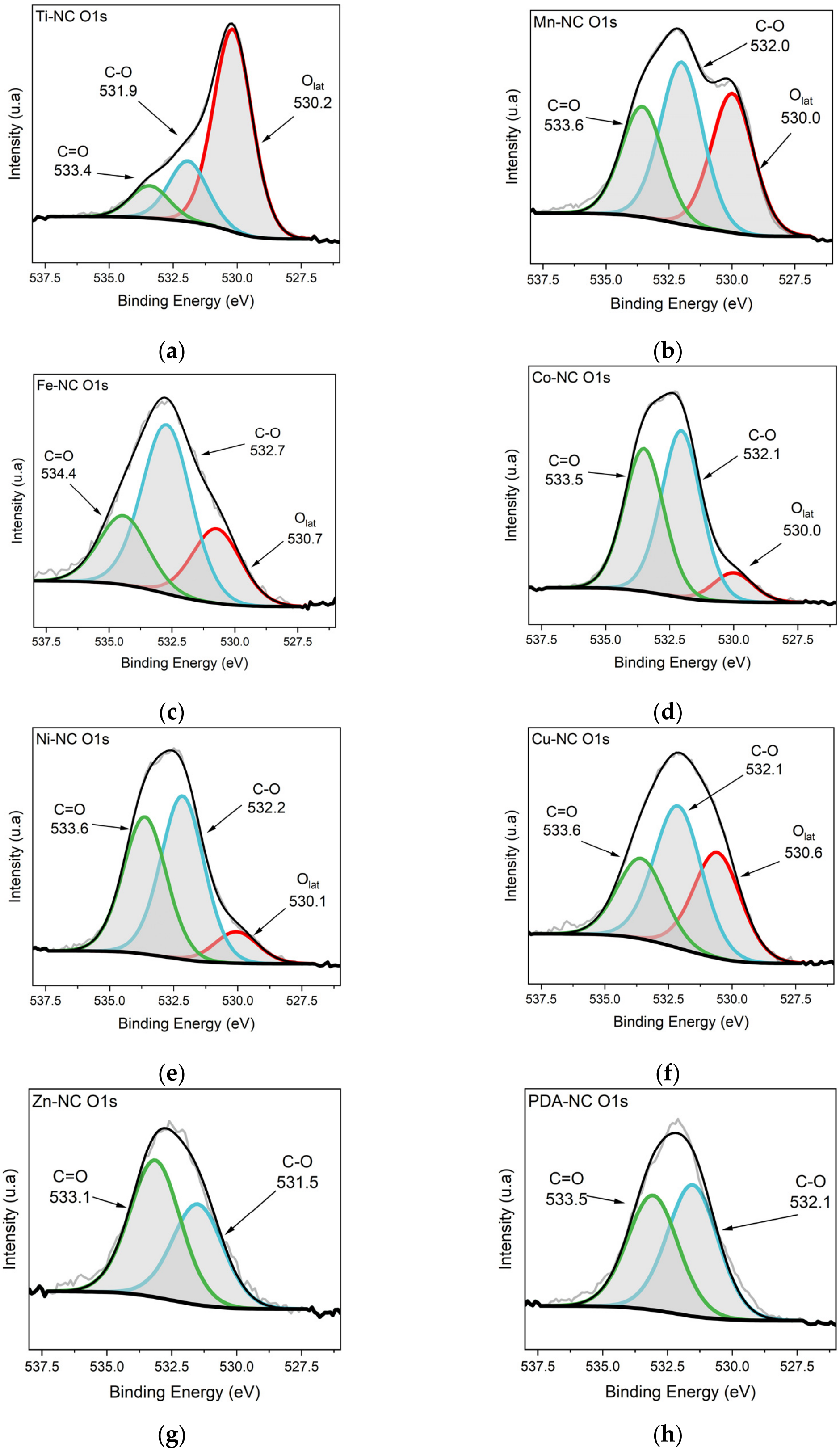

Figure S3. O 1s XPS spectra of (**a**) Ti-NC; (**b**) Mn-NC; (**c**) Fe-NC; (**d**) Co-NC; (**e**) Ni-NC; (**f**) Cu-NC; (**g**) Zn-NC; (**h**) PDA-NC

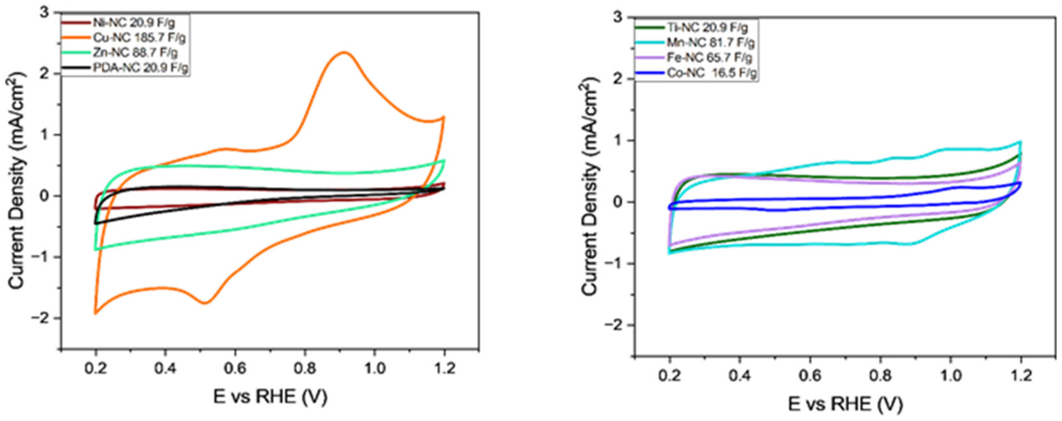

Figure S4. CV profile of the as-synthesized electrocatalysts measured in 0.1 M NaOH at a scan rate of 20 mV s^{-1}
